# Analytical Evaluation of Three Portable Blood Glucose Meters for Human Use in Dogs

**DOI:** 10.3390/vetsci12050452

**Published:** 2025-05-09

**Authors:** José Lucas Xavier Lopes, Taís Bock Nogueira, Luana Rodrigues, Vitória Strzeleski Wodzik, Denise Iparraguirre da Silva, Bruna dos Santos Machado, Álan Gomes Pöppl

**Affiliations:** 1 Veterinary Sciences Post-Graduate Program (PPGCV), Federal University of Rio Grande do Sul (UFRGS), Porto Alegre 91540-000, Brazil; jose.xavier@ufrgs.br (J.L.X.L.); taisbock2@gmail.com (T.B.N.); vetluanarodrigues@gmail.com (L.R.); vitoria.wodzik@ufrgs.br (V.S.W.); 2 Faculty of Veterinary, Federal University of Rio Grande do Sul (UFRGS), Porto Alegre 91540-000, Brazil; deniipa@gmail.com (D.I.d.S.); brunadsm@ufrgs.br (B.d.S.M.); 3 Department of Animal Medicine, Federal University of Rio Grande do Sul (UFRGS), Porto Alegre 91540-000, Brazil

**Keywords:** International Organization for Standardization 15197:2013, blood glucose, hypoglycemia, hyperglycemia, canine

## Abstract

Portable blood glucose meters (PBGMs) are essential for detecting glycemic disorders. In Brazil, veterinary devices are scarce, and human devices are used as an alternative. This study evaluated the accuracy of three human glucometers: Accu-Chek Guide^®^ (ACG), Accu-Chek Guide Me^®^ (ACGM) (Roche), and EcoCheck^®^ (EC) (Eco Diagnóstica). A total of 419 glycemic samples were collected and compared to the glucose oxidase method (GOM), which is considered to be the gold standard. Despite a strong positive correlation compared to the GOM (ACG r = 0.96; ACGM r = 0.9; EC r = 0.89; *p* < 0.0001), when evaluated by the criteria defined by ISO 15197:2013 for analytical precision, no PG obtained 99% of the readings within the range of +/− 15 for glucose levels < 100 mg/dL and +/− 15% for glucose levels > 100 mg/dL (ACG = 57.9%, ACGM = 47.9%, and EC = 28.4%). ACG was the only PG that fully met the clinical precision criteria according to ISO 15197:2013, with 100% of its measurements in Zones A+B of the error grid. Despite analytical inaccuracy, the ACG PBGM proved to be the best among the devices tested in this study for use in dogs with whole blood samples, not generating any negative impact on animal health.

## Simple Summary

Portable blood glucose meters (PBGMs) provide a convenient means of obtaining near-instantaneous glycemic measurements, serving as a valuable alternative in situations where laboratory resources are unavailable. This study evaluated the accuracy of three human-use PGMs in measuring glycemia in dogs, comparing their results to the gold-standard method. The goal was to ascertain whether these devices could be deemed reliable for veterinary application and whether treatment decisions based on these measurements could impact the health of the animals, particularly given the scarcity of veterinary-specific devices in Brazil and the rapid turnover of human-use devices in the Brazilian market.

## 1. Introduction

Glycemic measurement serves as an essential tool for diagnosing and monitoring various diseases in both humans and domestic animals [1]. Most laboratories utilize automated analyzers, primarily based on hexokinase or glucose oxidase reactions, which are regarded as reference methods (RMs) for glycemic assessment [2,3].

In situations where RMs for glycemic measurement are not accessible, particularly in emergencies, portable blood glucose meters (PBGMs) become invaluable. They provide immediate glycemic values, facilitating rapid clinical decisions, such as diagnosing diabetes mellitus (DM) or determining the necessity for glucose supplementation in a hypoglycemic animal [4,5]. DM is a common clinical diagnosis in Brazil due to the overrepresentation of the progesterone-related form [6]. The PBGM’s use may not only help provide a quick diagnosis but it is also quite inexpensive compared to a reference method, often performed in laboratories outside the clinical setting. Also, glycemic evaluation by the owners at home is a valuable tool during insulin therapy adjustments and long-term monitoring [7]. Notwithstanding, serial blood glucose curves in diabetic dogs are somewhat difficult to interpret due to the day-to-day variability, without being further impaired by a PBGM’s inaccuracy [8].

Historically, PBGMs have been developed for measuring capillary glycemia in humans, with limited options available exclusively for veterinary use. As a result, veterinarians often resort to using PBGMs designed for humans on dogs and cats. One notable example is the AlphaTrak2^®^, a glucose meter recognized for its accuracy in measuring glycemic levels in canines and felines worldwide, although it is unavailable in Brazil. Meanwhile, a diverse array of human-use PBGMs are readily accessible, with new devices frequently entering the market and others being phased out, leading to significant turnover. However, it is crucial to evaluate the analytical accuracy and precision of these devices before incorporating them into routine veterinary clinical practice [7,9,10].

Thus, the objective of this study was to evaluate the analytical accuracy and clinical precision of three human-use PBGMs for determining glycemia in dogs using whole blood samples.

## 2. Materials and Methods

### 2.1. Patients

This study was conducted with canine patients seen in the routine of the Veterinary Endocrinology and Metabolism Service (SEMV—PetEndocrine^®^) at the Veterinary Teaching Hospital of the Federal University of Rio Grande do Sul (HCV/UFRGS) in Brazil. The study included patients with a clinical indication for blood sampling, specifically for glycemic evaluation during their consultation, resulting in 69 dogs. Some of these patients participated at different time points throughout the study. This population included 33 (47.8%) dogs with DM, 28 (40.6%) dogs with Cushing’s syndrome, 3 (4,3%) dogs without endocrine diseases being evaluated for check-up, 2 (2.9%) dogs with hypothyroidism, and another 3 dogs diagnosed with alopecia X, primary hyperlipidemia, and insulinoma (1.4% each).

At each evaluation, dogs underwent a complete blood count (CBC) as part of their overall health status evaluation and to evaluate their hematocrit (HT). Serum biochemical and hormonal variables were also performed according to the individual’s history. Importantly, the devices used in this research work adequately in a hematocrit range between 10 and 68%. None of the patients exhibited hematocrit values out of this range.

### 2.2. Blood Collection and Glycemia Determination

The samples were collected via the venous puncture of the jugular vein using a 5 mL syringe equipped with a 30 × 0.7 mm hypodermic needle during the dog’s clinical consultations in the endocrine service. Immediately after collection, the samples were aliquoted into three separate tubes (Vacutainer, BD^®^, Franklin Lakes, NJ, USA), each containing the following: (1) EDTA (for complete blood count analysis), (2) a tube without anticoagulant (for specific biochemical determinations as needed), and (3) EDTA with sodium fluoride for glycemia measurement using the enzymatic colorimetric glucose oxidase method, performed with an automatic spectrophotometer (CM 200^®^, Wiener Lab Group, Rosario, Argentina). To transfer the samples into the respective tubes, the syringe needle was detached, and after removing the caps, the blood was gently injected into each tube, allowing it to flow down the inner wall. The samples in the EDTA tubes were then homogenized. Additionally, a small final aliquot of whole blood was retained in the syringes for immediate glycemic measurement with the tested PBGMs at room temperature.

### 2.3. Technical Information of the Tested PBGMs

The devices utilized in this study were the Accu-Chek Guide^®^ (ACG) and Accu-Chek Guide ME^®^ (ACGM), both produced by Roche Diagnostics, Basel, Switzerland, as well as the EcoCheck^®^ (EC) device from Eco Diagnóstica in Nova Lima, Brazil. Each device requires a sample volume of 0.6 µL for glycemic readings and has an operational range of 10–600 mg/dL. Readings outside this range are indicated on the devices as “Low” or “High”, respectively. According to the manufacturers, the ACG and ACGM devices are not influenced by hematocrit variations within the range of 10% to 65%, while the EC device is free from hematocrit interference within a range of 20% to 68%.

All devices were assessed for their analytical accuracy according to the international organization for standardization [11]. To be deemed accurate, the devices must demonstrate a maximum variation of ±15 mg/dL when glycemia is ≤100 mg/dL, or ±15% when glycemia is ≥100 mg/dL, achieving this in at least 95% of measurements compared to the reference method [11]. Furthermore, clinical precision was evaluated using the Parkes error grid, which is divided into five zones: A (clinically accurate), B (altered clinical action, but with no or minimal effect on clinical outcome), C (altered clinical action likely to affect the clinical outcome), D (altered clinical action with considerable medical risk), and E (altered clinical action with potentially dangerous consequences) [12]. For a glucose meter to be recognized as accurate, ISO 2013 mandates that 99% of glycemic readings fall within Zones A and B [11]. The coefficient of variation (CV%) for each device was also calculated based on three repeated measurements of the same blood sample. Samples were categorized as hypoglycemic if the values were <60 mg/dL, normoglycemic if they fell between 60 and 116 mg/dL, and hyperglycemic if they exceeded 116 mg/dL [13,14].

### 2.4. Statistical Analyses

Statistical analysis was conducted using GraphPad Prism software (version 6.05, San Diego, CA, USA). The Pearson linear correlation coefficient was determined between the results obtained with the different glucose meters and the RM. Values of *p* < 0.05 were considered statistically significant. Additionally, the difference between the results determined by the PBGMs and the RM was represented through Bland–Altman plots [15]. The results were distributed with each glucose meter in the Parkes error grid using the BD Error Grid (version 1.0) spreadsheet for Excel on Windows [12].

## 3. Results

The sample size tested with each glucose meter was not uniform, as the study initially concentrated on the analytical evaluation of the EC device (*n* = 204), followed by the inclusion of the ACG device (*n* = 69). However, the ACG was discontinued in Brazil during the study and was subsequently replaced by the ACGM device (*n* = 146). In total, 419 samples were assessed across the three glucose meters. Among these, 34 samples (8.1%) were classified as hypoglycemic (mean = 36.7 ± 14.15 mg/dL), 181 samples (43.1%) were classified as normoglycemic (mean = 88 ± 14.12 mg/dL), and 204 samples (48.6%) were classified as hyperglycemic (mean = 317.7 ± 126.11 mg/dL).

**Figure 1:**
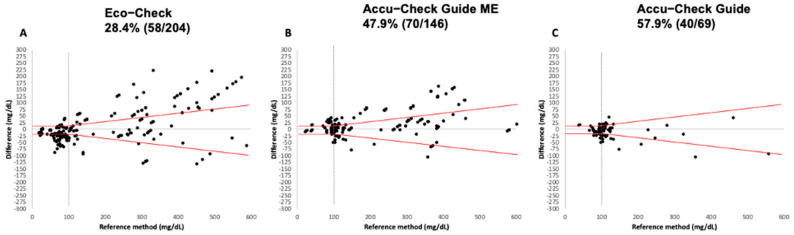
The Bland–Altman scatter plots for each PBGM are illustrated above. The X-axis (the horizontal line at the bottom of the graph) represents the glycemia values obtained by the reference method (RM), while the Y-axis (the vertical line) shows the difference between the glycemic value obtained by the PBGM and that from the RM. The red lines denote the limits established by ISO 15197:2013 for classifying device accuracy. The gray line that intersects the red lines vertically indicates the trivial glycemic threshold of 100 mg/dL. According to ISO 2013, the devices must adhere to a maximum variation limit for PBGMs to be deemed accurate. The plots correspond to the following devices: (**A**) Eco Check; (**B**) Accu-Chek Guide ME, and (**C**) Accu-Chek Guide.

None of the three devices met the analytical accuracy criteria established by ISO 15197:2013 (see Figure 1). For the EC device, only 28.4% (Figure 1A) of the readings fell within the acceptable variation range, while for the ACGM and ACG devices, this figure was 47.9% (Figure 1B) and 57.9% (Figure 1C), respectively.

**Figure 2:**
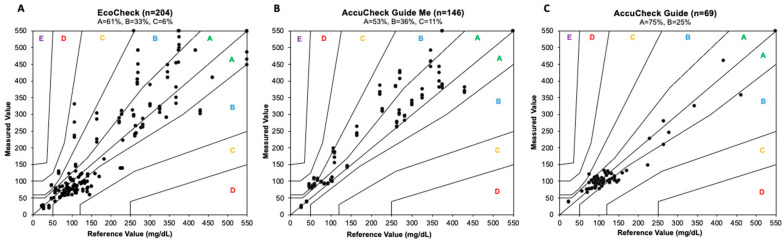
Error grids of the PBGMs utilized in the study. Zone A indicates sufficient accuracy with no clinical impact; Zone B suggests a change in management with no impact on the outcome; Zone C involves a change in management with some impact on the outcome; Zone D represents a change in management with a potential considerable medical risk; and Zone E reflects dangerous consequences for the patient. To be deemed clinically accurate, a glucose meter must demonstrate that at least 99% of glycemia readings fall within Zones A and B. The Accu-Chek Guide was the only device to meet this criterion completely, achieving 100% glycemia readings within the reference ranges. In contrast, the PBGMs EC and ACGM only reached 94% and 89%, respectively, and were therefore not classified as clinically accurate.

In terms of clinical accuracy, neither the EC nor the ACGM devices satisfied the ISO criteria, with only 94% of measurements falling within Zones A and B (61% in Zone A and 33% in Zone B) for the EC device and 89% (53% in Zone A and 36% in Zone B) for the ACGM. In contrast, the ACG device emerged as the most accurate PBGM in this study, achieving 100% of measurements within Zones A and B, with 75% in Zone A and 25% in Zone B (refer to Figure 2A–C).

**Figure 3:**
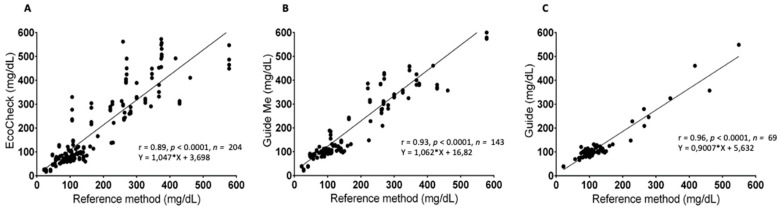
Pearson correlation graph illustrating the relationship between the evaluated PBGMs and the reference method (RM): (**A**) EcoCheck; (**B**) Accu-Chek Guide ME; (**C**) Accu-Chek Guide. This graph exhibits a positive correlation at varying levels between the assessed PBGMs and the RM, particularly highlighting the Accu-Chek Guide (ACG), which showed a correlation with the RM of r = 0.96, indicating a strong agreement with the RM.

The three PBGMs exhibited a notable positive correlation, as indicated by Pearson’s correlation coefficient (Figure 3). Specifically, for the ACG PBGM, the correlation coefficient was r = 0.96 (95% CI = 0.93 to 0.97; *p* < 0.0001). The ACGM PBGM demonstrated a coefficient of r = 0.93 (95% CI = 0.90 to 0.95; *p* < 0.0001), while the EC PBGM showed a coefficient of r = 0.89 (95% CI = 0.87 to 0.92; *p* < 0.0001).

Regarding the coefficient of variation (CV%), no significant difference was observed among the CVs of the different PBGMs evaluated (*p* = 0.077), despite the wide variability documented in some readings of the EC device, as shown in Table 1.

## 4. Discussion

PBGMs play a crucial role in small animal clinical practice. However, in certain countries, the absence of devices specifically designed for canine and feline species makes it necessary to use human devices as an alternative. This is acceptable as long as these devices are validated for use in animals or at least do not result in erroneous decision-making regarding patient management [1,16,17,18,19,20]. The validity of various human-use PBGMs for application in dogs is a topic of frequent study [21,22,23].

The validation of various human-use point-of-care PBGMs for application in dogs is a subject of frequent research [1,4,16,17,18,19,20,21,22,23]. A study comparing the ACG device with the One Call Plus II^®^ (OCP) claimed the former as more accurate and precise [23], despite differences with our study in statistical analysis and glycemic ranges. Despite the small number of samples included in the evaluation of the ACG in our study, especially above the actual cutoff for canine DM diagnosis (>200 mg/dL) [24], our results reinforce this device as a relatively good choice for veterinary use in dogs. To the authors’ knowledge, no other studies have assessed the analytical accuracy of the EcoCheck^®^ (EC) and Accu-Chek Guide ME^®^ (ACGM) glucose meters. Despite the ACG device being off the market in Brazil, its superior analytical and clinical performance compared with its substitute (ACGM) warrants its continuous use, since its test strips are still available and are the same as those used with the newly released ACGM.

According to Gerber and Freeman [9], several factors can interfere with glycemic measurement, including lipemia, plasma pigments such as bilirubin, blood pH, partial oxygen pressure (PO2), and hematocrit levels. A limitation of our study was that, among these variables, only the patient’s hematocrit was systematically assessed across all samples. Despite other blood tests (serum biochemistry and serum hormone concentration) being eventually performed according to individual cases, mixed clinical pictures were present among the study participants and may have ultimately biased the results. However, this limitation is amenable in a clinical setting with real clinical cases (i.e., not in experimental dogs with induced hypo or hyperglycemia).

Documenting hematocrit levels and understanding the interference-free range of the PBGM is essential, as anemic patients may display overestimated glycemic readings, while those with elevated hematocrit levels could show underestimated readings. These discrepancies arise from the differential plasma contact with the reagent strip, leading to either increased or decreased measurement accuracy [14,18,25,26]. Even so, given the risk hypoglycemia brings to the pet [13], and the burden of a hypoglycemic crisis on the owners [27], it may be considered less problematic that a given PBGM reads lower than the real glucose value than the opposite.

The findings of this study reveal that, although there is a strong correlation between the results obtained from the human-use PBGMs ACG, ACGM, and EC compared to the laboratory method, none of these devices entirely satisfied the analytical accuracy recommendations outlined in ISO 15197:2013. The significance and relevance of portable glucose meters in clinical practice are clear. However, selecting these devices should not be carried out indiscriminately or haphazardly. The frequent introduction of new models and the phasing out of older ones contribute to a rapidly evolving market. Consequently, this study underscores the necessity of rigorously assessing both the analytical and clinical accuracy of the available portable blood glucose meters (PBGMs), particularly those intended for human use, given the limited availability of veterinary-specific options in Brazil.

This ISO standard has served as a foundational reference for research assessing the clinical and analytical accuracy of human glucose meters, and it is commonly utilized in veterinary medicine studies to evaluate these parameters [11]. The research conducted by Brito-Casillas et al. [16] assessed the accuracy of nine human-use glucose meters in dogs, adhering to this standard. This study was pioneering in its adoption and promotion of the standard within veterinary medicine, thus setting a benchmark for future research aimed at examining the accuracy and precision of human glucose meters in animals [11,16].

In terms of clinical accuracy, only the ACG device met the necessary criteria to be classified as clinically precise according to the Parkes error grid, with 100% of its measurements falling within Zones A and B. In contrast, neither of the other two evaluated devices fulfilled the minimum requirements established by ISO 15197:2013. This indicates that making therapeutic decisions based on glycemic results from the ACGM and EC devices could potentially lead to inappropriate actions for patients, although errors categorized in Zone C of the error grid are considered low-risk [12].

The evaluation of three glucose meters revealed a significant positive correlation, as indicated by Pearson’s correlation coefficient. The correlation was classified as very strong (>0.9) for the PBGMs ACG (r = 0.96) and ACGM (r = 0.93). In contrast, the correlation for the EC glucose meter was categorized as merely “strong” (r = 0.89). It is worthwhile that the correlation coefficient evaluated in isolation may create a false impression of accuracy. In this way, the ISO 15197:2013 recommendations to evaluate a PBGM’s accuracy are warranted. Additionally, an analysis of the coefficient of variation (CV%) showed that, on average, although there were no significant differences among the three devices tested, the readings from the EC PBGM demonstrated the highest dispersion index, with differences reaching up to 36% among readings of the same sample. Understanding the CV% of PBGMs is crucial in clinical practice, as glycemic readings are frequently taken as single-point measurements, and results considered to be normal are seldom confirmed subsequently [28].

## 5. Conclusions

In our research, none of the glucose meters met the ISO 15197:2013 standards, resulting in their analytical inaccuracy. However, the Accu-Chek Guide^®^ successfully fulfilled the required specifications for clinical accuracy, making it the superior choice among the devices tested for routine glycemic monitoring in dogs, delivering prompt and reliable results. Nonetheless, its discontinuation in the Brazilian market indicates that its successor, the Accu-Chek Guide ME, emerges as the most suitable option among the PBGMs evaluated.

## Figures and Tables

**Table 1:** The coefficients of variation (CV%) for the evaluated portable blood glucose meters are presented. Mean CV% values, along with 95% confidence intervals (CI95%) and the range of minimum to maximum CV% for the devices—EcoCheck^®^ (EC), Accu-Chek Guide^®^ (ACG), and Accu-Chek Guide ME^®^ (ACGM)—are included. The table emphasizes the precision variability among the devices, revealing that EC has the highest variability range, whereas the ACG and ACGM demonstrate relatively lower and more consistent ranges of variability.

PBGM	Mean CV%	CV% CI 95%	CV% Minimum–Maximum
EC	6.09	4.06−9.73	0.9−36
ACG	3.02	2.18−3.86	0.21−9.39
ACGM	2.4	1.61−4.28	0.8−5.5

## Data Availability

The raw data supporting the conclusions of this article will be made available by the authors on request.

## Abbreviations

The following abbreviations are used in this manuscript:

PBGM	Portable blood glucose meters
ACGM	Accu-Chek Guide Me^®^
EC	EcoCheck^®^
ACG	Accu-Chek Guide^®^
GOM	Glucose oxidase method
ISO	International Organization for Standardization
RM	Reference method
CBC	Complete blood count
HT	Hematocrit
CV%	Coefficient of variation
OCP	One Call Plus II^®^
PO2	Partial oxygen pressure

## References

[1] Cohn L.A., McCaw D.L., Tate D.J., Johnson J.C. (2000). Assessment of five portable blood glucose meters, a point-of-care analyzer, and color test strips for measuring blood glucose concentration in dogs. J. Am. Vet. Med. Assoc..

[2] Sonagra A.D., Zubair M., Motiani A. (2024). Hexoquinase Method. Stat Pearls.

[3] Trinder P. (1969). Determination of glucose in blood using glucose oxidase with an alternative oxygen acceptor. Ann. Clin. Biochem..

[4] Jamaluddin F.A., Gunavathy M., Yean C.Y., Thevarajah M. (2017). Variability of point-of-care testing blood glucometers versus the laboratory reference method in a tertiary teaching hospital. Asian Biomed..

[5] Casella M., Hässig M., Reusch C.E. (2005). Home-monitoring of blood glucose in cats with diabetes mellitus: Evaluation over a 4-month period. J. Feline Med. Surg..

[6] Pöppl A.G., Lopes J.L.X., Nogueira T.B., da Silva D.I., Machado B.D.S. (2024). Progesterone-Related Diabetes Mellitus in the Bitch: Current Knowledge, the Role of Pyometra, and Relevance in Practice. Animals.

[7] Behrend E., Holford A., Lathan P., Rucinsky R., Schulman R. (2018). AAHA Diabetes Management Guidelines for Dogs and Cats. J. Am. Anim. Hosp. Assoc..

[8] Fleeman L.M., Rand J.S. (2003). Evaluation of day-to-day variability of serial blood glucose concentration curves in diabetic dogs. J. Am. Vet. Med. Assoc..

[9] Gerber K.L., Freeman K.P. (2016). ASVCP guidelines: Quality assurance for portable blood glucose meter (glucometer) use in veterinary medicine. Vet. Clin. Pathol..

[10] Shapiro B., Savage P.J., Lomatach D., Gniadek T., Forbes R., Mitchell R., Hein K., Starr R., Nutter M., Scherdt B. (1981). A comparison of accuracy and estimated cost of methods for home blood glucose monitoring. Diabetes Care.

[11] (2013). In Vitro Diagnostic Test Systems—Requirements for Blood-Glucose Monitoring Systems for Self-Testing in Managing Diabetes Mellitus.

[12] Parkes J.L., Slatin S.L., Pardo S., Ginsberg B.H. (2000). A New Consensus Error Grid to Evaluate the Clinical Significance of Inaccuracies in the Measurement of Blood Glucose. Diabetes Care.

[13] Idowu O., Heading K. (2018). Hypoglycemia in Dogs: Causes, Management, and Diagnosis. Can. Vet. J..

[14] Suchowersky N.D., Carlson E.A., Lee H.P., Behrend E.N. (2021). Comparison of Glucose Concentrations in Canine Whole Blood, Plasma, and Serum Measured with a Veterinary Point-of-Care Glucometer. J. Vet. Diagn. Investig..

[15] Bland J.M., Altman D.G. (1986). Statistical Methods for Assessing Agreement Between Two Methods of Clinical Measurement. Lancet.

[16] Brito-Casillas Y., Figueirinhas P., Wiebe J.C., López-Rios L., Pérez-Barreto D., Mélian C., Wägner A.M. (2014). ISO-Based Assessment of Accuracy and Precision of Glucose Meters in Dogs. J. Vet. Intern. Med..

[17] Johnson B.B., Fry M.M., Flatland B., Kirk C.A. (2009). Comparison of a Human Portable Blood Glucose Meter, Veterinary Portable Blood Glucose Meter, and Automated Chemistry Analyzer for Measurement of Blood Glucose Concentrations in Dogs. J. Am. Vet. Med. Assoc..

[18] Dobromylskyj M.J., Sparkes A.H. (2010). Assessing Portable Blood Glucose Meters for Clinical Use in Cats in the United Kingdom. Vet. Rec..

[19] Kang M.H., Kim D.H., Jeong I.S., Choi G.C., Park H.M. (2016). Evaluation of Four Portable Blood Glucose Meters in Diabetic and Non-Diabetic Dogs and Cats. Vet. Q..

[20] Wess G., Reusch C. (2000). Assessment of Five Portable Blood Glucose Meters for Use in Cats. Am. J. Vet. Res..

[21] Ismail-Hamdi S., Romdane M.N., Romdhane S.B. (2021). Comparison of a human portable blood glucose meter and automated chemistry analyzer for measurement of blood glucose concentrations in healthy dogs. Vet. Med. Sci..

[22] Dos Santos M.A.B., Vargas A.M., Rosato P.N., Andrade C.G., Martins C.M., Petri G. (2022). Evaluation of three human-use glucometers for blood glucose measurement in dogs. Vet. Med. Int..

[23] Souza K.T.R.d., Monteiro L.F., Knackfus F.B., Monteiro L.M.V.W. (2021). Study of data obtained in the evaluation of two portable glucometers for human use in dogs. Multidiscip. Sci. J..

[24] Niessen S.J.M., Bjornvad C., Church D.B., Davison L., Esteban-Saltiveri D., Fleeman L.M., Forcada Y., Fracassi F., Gilor C., Hanson J. (2022). Agreeing Language in Veterinary Endocrinology (ALIVE): Diabetes mellitus—A modified Delphi-method-based system to create consensus disease definitions. Vet. J..

[25] Mori A., Lee P., Yokoyama T., Oda H., Saeki K., Miki Y., Nozawa S., Azakami D., Momota Y., Makino Y. (2011). Evaluation of artificial pancreas technology for continuous blood glucose monitoring in dogs. J. Artif. Organs..

[26] Nichols J.H., Howard C., Loman K., Miller C., Nyberg D., Chan D.W. (1995). Laboratory and bedside evaluation of portable glucose meters. Am. J. Clin. Pathol..

[27] Niessen S.J., Powney S., Guitian J., Niessen A.P., Pion P.D., Shaw J.A., Church D.B. (2012). Evaluation of a quality-of-life tool for dogs with diabetes mellitus. J. Vet. Intern. Med..

[28] Vieira S. (2016). Introdução à Bioestatística.

